# Effect of Cross-Linked Alginate/Oil Nanoemulsion Coating on Cracking and Quality Parameters of Sweet Cherries

**DOI:** 10.3390/foods10020449

**Published:** 2021-02-18

**Authors:** Camilo Gutiérrez-Jara, Cristina Bilbao-Sainz, Tara McHugh, Bor-Sen Chiou, Tina Williams, Ricardo Villalobos-Carvajal

**Affiliations:** 1 Food Engineering Department, Universidad del Bío-Bío, P.O. Box 447, Av. Andrés Bello 720, Chillán 3800708, Chile; camilo.gtz.j@gmail.com; 2 Western Regional Research Center, United States Department of Agriculture, Albany, CA 94710, USA; cristina.bilbao@usda.gov (C.B.-S.); tara.mchugh@ars.usda.gov (T.M.); bor-sen.chiou@usda.gov (B.-S.C.); tina.williams@ars.usda.gov (T.W.)

**Keywords:** sweet cherry, nanoemulsion coating, cracking, fruit quality, nutraceutical value, crosslinking

## Abstract

The cracking of sweet cherries causes significant crop losses. Sweet cherries (cv. Bing) were coated by electro-spraying with an edible nanoemulsion (NE) of alginate and soybean oil with or without a CaCl_2_ cross-linker to reduce cracking. Coated sweet cherries were stored at 4 °C for 28 d. The barrier and fruit quality properties and nutritional values of the coated cherries were evaluated and compared with those of uncoated sweet cherries. Sweet cherries coated with NE + CaCl_2_ increased cracking tolerance by 53% and increased firmness. However, coated sweet cherries exhibited a 10% increase in water loss after 28 d due to decreased resistance to water vapor transfer. Coated sweet cherries showed a higher soluble solid content, titratable acidity, antioxidant capacity, and total soluble phenolic content compared with uncoated sweet cherries. Therefore, the use of the NE + CaCl_2_ coating on sweet cherries can help reduce cracking and maintain their postharvest quality.

## 1. Introduction

The sweet cherry fruit has a high nutritional value, mainly due to its high antioxidant capacity associated with ascorbic acid, carotenoids, and phenolic compounds [1]. The phenolic compounds in sweet cherries play a protective role against oxidative stress, ultraviolet radiation, and free radical damage [2]. However, the rapid deterioration of sweet cherries after harvest often leads to quality loss. More research is needed to develop novel strategies to prevent or reduce postharvest deterioration [3].

The cracking of sweet cherries caused by rain during the harvest period is the most important source of crop loss in the industry [4]. Rainfall, high humidity, high temperature, rootstock type, crop load, soil moisture levels, and irrigation management are some of the main factors that affect sweet cherry cracking [5]. As for the development of cracks on the skin of the fruits, the main mechanism proposed is related to the increase in turgor pressure caused by water absorption during and after rain. The two main routes of water absorption occur through the fruit surface [6] and/or the roots of the tree [7]. Various strategies have been used to reduce cracking. These include the use of plastic rain shields; adequate irrigation management; the application of calcium salts; and, more recently, the use of protective waterproofing agents [5]. The use of these technologies can reduce the severity of the damage. However, the degree of effectiveness widely varies among seasons, cultivars, and geographic locations. Some strategies, such as plastic rain shields, have high implementation costs [8]. 

The use of edible coatings is an alternative strategy that has emerged in recent decades [9,10]. This approach could be used to decrease the cracking phenomena of sweet cherries and extend their postharvest shelf life. Some of these edible coatings include chitosan [11], Aloe vera gel [12], and Semperfresh [13,14]. 

Alginate is another film-forming material that has been used as a thickening agent, gelling agent, and stabilizer in a variety of food emulsions [15]. It is a natural polysaccharide that is extracted from brown seaweed (Phaeophyceae) and comprises the two uronic acids, β-D-manuronic and α-L-guluronic. Sodium alginate consists of block polymers of sodium poly-L-guluronate, sodium poly-D-mannuronate, and alternate sequences of both sugars [16]. It has been effective in maintaining the postharvest quality of tomatoes [17], peaches [18], and sweet cherries [1]. However, to the best of our knowledge there are no previous studies on using alginate-based coatings to prevent sweet cherry cracking. 

The cross-linking of the alginate film surface can be used to improve its mechanical and barrier properties because the film disintegrates when subjected to high humidity conditions due to its hydrophilic nature [19]. Cross-linking methods usually include drying, heating, ultraviolet (UV) irradiation, and chemical methods [20]. The chemical cross-linking method for sodium alginate involves the ionic interaction between polymer chains and multivalent ions to form ionomers. This improves their water barrier properties, mechanical strength, cohesiveness, and rigidity [10,21]. 

Another way to improve the water vapor barrier properties of the film involves adding lipids and nanofillers to form composite or nanocomposite films [22,23,24,25]. Smaller lipid globules and a more homogeneous distribution of the oil droplets in the films generally lead to better water vapor barrier properties [26]. 

Therefore, the aim of this study was to apply a nanoemulsion (soybean oil with alginate solution) on sweet cherries with additional ionic cross-linking and evaluate its effect on the water barrier properties of the sweet cherry cuticle, such as fruit cracking, postharvest qualities, and nutraceutical values.

## 2. Materials and Methods

### 2.1. Plant Material and Experimental Design

Sweet cherries (*Prunus avium* L.) cv. Bing were randomly collected at the commercial maturity stage from the midsection of 10 trees grown under standard commercial practices on the same commercial farm located in Brentwood (CA, USA). Cultural practices were regularly implemented for all trees equally. Fruits were transported immediately to the laboratory and selected by color, size, and the absence of physical defects or decay. Subsequently, the fruits were randomly distributed in 3 batches of 111 fruits each prior to treatment. The first batch was treated with a nanoemulsion (NE). The second batch was treated with a nanoemulsion and CaCl_2_ solution (NE + CaCl_2_). The third batch was used as a control. The barrier properties were determined immediately after each coating treatment. The quality parameters of the fruits stored at 4 °C were evaluated weekly for 28 d.

### 2.2. Nanoemulsion Preparation

The alginate solution (1.0%, *w*/*v*) was prepared by dissolving sodium alginate in a 2.5% ethanol solution. Ethanol was used to decrease the surface tension of nanoemulsions [27]. Glycerol was added at 15% alginate mass and the alginate solution was stirred for 30 min. Tween 80 (1.0% *v*/*v*) and soybean oil (0.5% *v*/*v*) were added to the solution and homogenized at 11,000 rpm for 2 min with a rotor-stator homogenizer (Polytron 3000, Kinematica, Littau, Switzerland). These coarse emulsions were passed six times through a microfluidizer processor (model 110T, Microfluidics, Asheville, NC, USA) at 200 MPa to obtain the nanoemulsions. The composition of the nanoemulsion and the process variables were selected on the basis of a prior study [28]. 

#### Particle Size and Polydispersity Index (PdI) of Nanoemulsions

The average nanoemulsion particle size and polydispersity index (PdI) were determined by dynamic light scattering (DLS) with a Zetasizer Nano ZS laser diffractometer (Malvern Instruments Ltd., Westborough, MA, USA). Emulsion samples were diluted in ultrapure water to 10% of the original concentration, placed in a cuvette, and analyzed at 25 °C. The average particle size (z-average) value and PdI were recorded.

### 2.3. Coating Application

The nanoemulsion (NE) was sprayed for 30 s at 30 cm from the surface of the sweet cherries with a cordless 85 kV vector solo waterborne electrostatic gun applicator (ITW Ransburg, Toledo, OH, USA). A 3.0% calcium chloride solution was applied using the same spray system after a coating was formed on the sweet cherries. 

### 2.4. Microstructure

The microstructure of sweet cherry cross-sections was observed with a JEOL 7900F field emission scanning electron microscope (SEM) (JEOL, Kyoto, Japan) with a Quorum PP3010T cryo-system. First, the sweet cherries were cut parallel to the longitudinal axis with a scalpel. The sample was placed in the SEM sample holder and plunged into subcooled nitrogen (−210 °C). Afterward, the frozen sample was transferred to the cryo stage and freeze-fractured and gold-coated. The samples were viewed and photographed at 5 kV in the SEM.

### 2.5. Barrier Properties

#### 2.5.1. Cracking Index (CI)

The CI was determined using the method reported by Christensen [29]. For this purpose, sweet cherries harvested on the same day were selected based on size (22.3–24.9 mm), total soluble solids (20.03–20.24° Bx), firmness (3.70–3.78 N), water activity (0.964–0.978), and color (a * = 10.16–13.83). Thirty fruits with stems from each batch were submerged in distilled water at 20 °C for 5 h to induce cracking. The number of cracked fruits was counted at 1 h intervals. The CI was calculated using the formula expressed in Equation (1).

CI = ((5a + 4b + 3c + 2d + 1e)/(MPV)) × 100,(1)

where a, b, c, d, and e represent the number of cracked samples at each time interval and MPV is the maximum possible value (30 fruits × 5 h = 150).

#### 2.5.2. Resistance to Water Vapor Transfer (RWVT)

During the same day of harvest, coated and uncoated sweet cherries were placed in a desiccator at 75.65% relative humidity using a saturated sodium chloride solution. Fans were used to ensure a uniform relative humidity throughout the desiccator. The desiccator was placed in a thermostatic chamber maintained at 4 ± 1 °C. Sweet cherries were weighed at 2 h intervals at 0.0001 g accuracy. The Resistance to Water Vapor Transfer (RWVT) was estimated by the equation of the first modified Fick law as established by different authors [30,31]. Weight loss data were used under stationary conditions. The RWVT was calculated by Equation (2).

RWVT = [((a_w_ − %RH/100) PWV)/RT] × (*A*/*J*), (2)

where RWVT is the resistance to water vapor transfer (s/cm), a_w_ is the sweet cherry water activity determined with a water activity meter (Aqua LAB 4TE, Pullman, WA, USA), %RH is the relative humidity inside the desiccator, PWV is the water vapor pressure at the chamber temperature (mm Hg), R is the universal gas constant (3,464,629 mm Hg cm^3^/g K), T is the storage chamber temperature (K), A is the sweet cherry surface area at the beginning of the test (cm^2^), and J is the slope of the weight loss curve under stationary conditions (g/s).

### 2.6. Fruit Quality Parameters

#### 2.6.1. Weight Loss

Fruit weight loss was evaluated with a digital balance (Precisa XB 320M, Dietikon, Switzerland). Sweet cherries were individually weighed at the beginning of the experiment and on each sampling day (7, 14, 21, and 28). Weight loss was expressed as a percentage of the initial weight and calculated by Equation (3).

Weight loss (%) = ((W_o_ − W_f_)/W_o_) × 100 (3)

where W_o_ is the initial weight and W_f_ is the weight on the sampling day.

#### 2.6.2. Optical Properties

Color measurements were performed with a CR-300 colorimeter (Minolta Camera Co., Ltd., Osaka, Japan). The CIELAB parameters a*, b*, and L* were obtained with the D65 light source and an observation angle of 10° using the reflectance specular mode. The L* coordinate represented the lightness of the color (L* = 0 denoted black and L* = 100 denoted white), a* indicated the position between green and red (a* varied from −80 to +100), and b* was the extent of blueness/yellowness (b* varied from −50 to +70). 

The hue angle (h°) was calculated by Equation (4) as:

Hue angle = arctan (b*/a*). (4)

#### 2.6.3. Fruit Firmness

Mechanical tests were performed with a Texture Analyzer (TA-XT2i, Stable Microsystems Ltd., Surrey, UK) at room temperature using a puncture test. A probe (3 mm diameter stainless steel cylinder) with a trigger force of 5 N penetrated the sample to a depth of 8 mm at a speed of 1 mm s^−1^. Fruit firmness was measured as the maximum penetration force, and the results were expressed in newtons.

#### 2.6.4. Determination of Total Soluble Solids (TSS) and Titratable Acidity (TA)

For the TSS and TA tests, 5 g of sweet cherry tissue was homogenized in 25 mL of distilled water and filtered. The TSS was determined in the juice at 20 °C with a temperature-compensated LR-01 laboratory refractometer (Maselli Measurements Inc., Stockton, CA, USA). The TA was determined by titrating with 0.025 N of NaOH to pH 8.2 with a semi-automated titrator (Hanna Instruments, Woonsocket, RI, USA).

#### 2.6.5. Total Soluble Phenolic (TSP) Content

The TSP content was determined by the Folin–Ciocalteu method as described by Bilbao-Sainz et al. [32] with slight modifications. Samples (5 g) were homogenized with 20 mL of methanol with a Waring Laboratory Blender (Waring Commercial, Torrington, CT, USA) surrounded with dry ice for 1 min at medium speed. Samples were placed in tubes and stored for 20 to 72 h at 4 °C. Homogenates were centrifuged (rotor SA-600, Sorvall RC 5C Plus, Kendro Laboratory Products, Newtown, CT, USA) at 29,000× *g* for 15 min at 4 °C. Duplicate samples from each extract were used for the final analysis. A 150 μL aliquot of methanol extract was taken from the clear supernatant, diluted with 2400 μL of ultrapure water and 150 μL 0.125 mol L^−1^ Folin–Ciocalteu reagent, and incubated for 3 min at room temperature. The reaction was stopped by adding 300 μL of 0.5 mol L^−1^ Na_2_CO_3_ and the mixture was incubated for 25 min. Absorbance readings at 725 nm of clear supernatant samples were measured with a Shimadzu PharmaSpec UV-1700 spectrophotometer (Shimadzu Scientific Instruments, Inc., Columbia, MD, USA). A blank sample prepared with methanol was used as a control. The total amount of phenols was determined using a gallic acid standard curve and the results were expressed as milligrams of gallic acid equivalent (GAE) per gram of fresh weight (FW) of cherry purée. Three replicates were performed for each sample.

#### 2.6.6. Antioxidant Capacity (AC) Analysis

The AC analysis was adapted from Bilbao-Sainz et al. [32] with slight modifications. The same methanol extract from the TSP analysis was used for the AC analysis. Sample aliquots of 50 μL were taken from the clear supernatant (equivalent methanol volume as a control) and reacted with 2950 μL of 2,2-diphenyl-1-picrylhydrazyl (DPPH, 103.2 μmol L^−1^ in methanol; absorbance approximately 1.2 at 515 nm) in a covered shaker at room temperature. The samples were allowed to react until steady-state conditions were reached. The AC was calculated with the PharmaSpec UV-1700 spectrophotometer by measuring the decrease in sample absorbance at 515 nm compared with the blank methanol sample. The AC was reported as μg Trolox equivalent from a standard curve developed with Trolox (0–750 μg mL^−1^) and expressed as mg Trolox g^−1^ FW. Three replicates were performed for each sample.

#### 2.6.7. Total Anthocyanin Content

The total anthocyanin content was determined in duplicate with a PharmaSpec UV-1700 spectrophotometer (Shimadzu Scientific Instruments, Inc., Columbia, MD, USA) following the method reported by Serrano et al. [33]. Results were calculated by Equation (5) and expressed as milligrams 100 g^−1^ FW.

(5) $$\mathrm{Total}\;\mathrm{anthocyanins}=\frac{\left(\frac{\mathrm{ABS}}{\varepsilon \times l}\times \mathrm{MW}\times 1000\right)\times \left(\frac{V+W\times \rho }{1000}\right)}{W}\times 100$$

where ABS = absorbance of sample; ε = molar absorption coefficient (23,900 L mol^−1^ cm^−1^ for cyanidin-3-glucoside (cyd-3-glu)); l = path length in cm; MW = molecular weight (449.2 g mol^−1^ for cyd-3-glu); V = volume of dilution in mL; W = sample weight in g; ρ = specific weight (0.83 in mL g^−1^); and 100 = 100 g of FW.

### 2.7. Statistical Analysis

A completely randomized design was used in the experiments. Statistical analysis was performed with the Statgraphics Centurion XVII software (version 17.1 12, Statgraphics Technologies Inc., The Plains, VA, USA) by a one-way analysis of variance. Significant differences between means were determined by the least significant difference (LSD) test at the 5% significance level (*p* < 0.05).

## 3. Results and Discussion

### 3.1. Particle Size and Polydispersity Index (PdI) of Nanoemulsions

After six passes through the microfluidizer, an emulsion was obtained with an average droplet size of 376.89 ± 2.73 nm and a PdI of 0.36 ± 0.04. These results concur with findings reported by other authors [34,35], who indicate that the increased number of passes through a homogenization system produces a reduced particle size and a more homogeneous particle size distribution. Similar results have been found by Artiga-Artigas et al. [36]. They achieved an emulsion with a 261 nm particle size and 0.25 PdI by mixing sodium alginate with an oil-in-water emulsion before the homogenization process (five passes). The smallest droplet size found in that study could be related to the different emulsion compositions because they used Tween 20 as an emulsifier and did not add a plasticizer.

### 3.2. Microstructural Analysis

**Figure 1:**
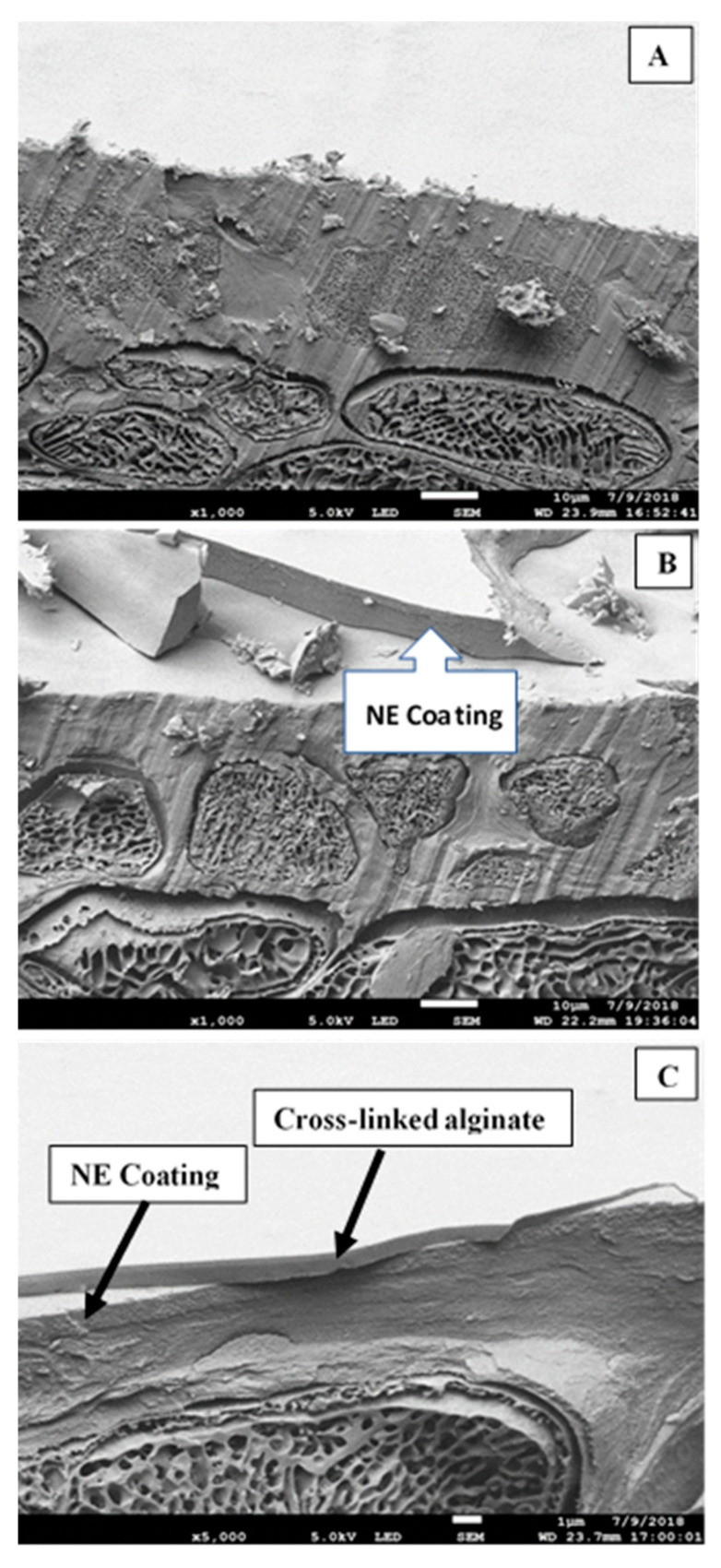
Cross-section micrographs of dried coatings on sweet cherry fruits. (**A**) Control, (**B**) nanoemulsion (NE), and (**C**) NE + CaCl_2_.

Figure 1 shows the cross-sections of fresh sweet cherry (A), the NE coating (B), and NE + CaCl_2_ coating (C) on the sweet cherry surfaces. The micrograph of the uncoated sweet cherry surface shows a layer of the cuticle membrane over a layer of the regular epidermal cells, similar to the findings reported by Bargel et al. [37]. The layer of epidermal cells can be observed in all three micrographs. Subepidermal cells increased in size below the layer of epidermal cells because they were located farther away from the surface. 

There was a continuous layer of NE coating on the sweet cherry surface (Figure 1B) as a result of the good adhesion of the NE. This adhesion could be attributed to the low surface tension of the coating formation solution because Tween 80 [38] and ethanol [27] were added. Meanwhile, the sweet cherries coated with NE + CaCl_2_ showed two layers. One layer was the NE on the cuticular membrane and the other was more compact and corresponded to the alginate cross-linked with calcium ions (arrows in Figure 1C). This second layer could reinforce the barrier properties of the cuticular membrane in sweet cherries.

### 3.3. Barrier Properties

#### 3.3.1. Cracking Index (CI)

**Figure 2:**
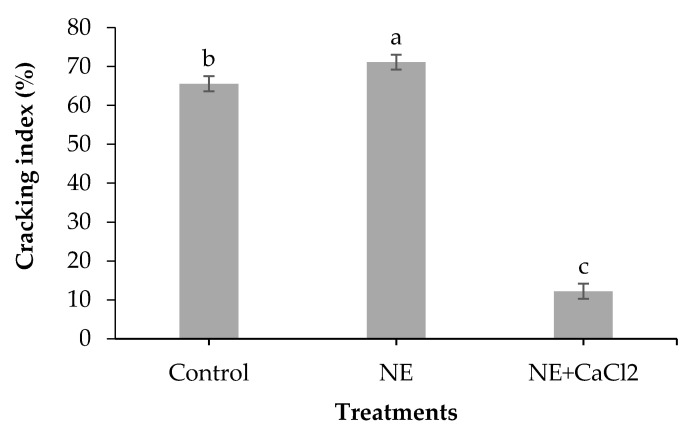
Effect of coating sweet cherries with nanoemulsion (NE) and nanoemulsion plus CaCl_2_ (NE + CaCl_2_) on the laboratory-induced cracking of sweet cherries. (a–c) Different letters indicate significant differences between treatments (*p* < 0.05).

Figure 2 shows the CI of coated and uncoated sweet cherries. The NE, which was expected to provide protection against water absorption by cherries and reduce their cracking, had the opposite effect and its application significantly increased the percentage of cracked sweet cherries (71.1%) compared with uncoated sweet cherries (65.6%). This effect could be due to the dissolution of the cuticular waxes by the Tween 80 emulsifier in the NE formulation, resulting in the increased water permeability of the cuticle [39]. This higher cuticle permeability could have induced the water diffusion inside the fruit, causing a localized burst of the cells that led to cracking [6].

Adding calcium ions to the NE coating dramatically increased the cracking tolerance and the percentage of cracked sweet cherries decreased from 65.5% to 12.2% (53.3% reduction). This result could be attributed to three different combined mechanisms. The first could be related to the decrease in osmotic potential on the fruit surface produced by the presence of calcium ions that did not react with the alginate [40]. Other authors mention that incorporating calcium ions on the surface of sweet cherries increased their cracking tolerance because of the decreased osmotic potential [41,42]. The second mechanism could involve strengthening the cuticular wax layer by Ca^2+^, hardening the cell walls to tolerate greater osmotic pressure before rupturing [43]. The third mechanism could be associated with the formation of a more compact and insoluble cross-linked alginate layer (Figure 1C) that increased the hydrophobicity of the sweet cherry surface [10,21]. This could have decreased the water diffusion from the outside to the inside of the sweet cherries, thus reducing the cracking percentage.

Other researchers have studied the application of hydrophobic coatings in the preharvest stage to reduce rain cracking in sweet cherries. Torres et al. [44] applied RainGard (mixture of fatty acids and vegetable oil) three times on cherry trees and reported 40.5%, 40.0%, and 52.0% reductions in rain cracking at harvest for Bing, Sweetheart, and Van cherries, respectively. They indicated that the coating waterproofed the surface of the cherries and acted as a filler for the micro-cracks in the cuticle. The application of cellulose nanofiber-based hydrophobic coatings (Innofresh) to Sweetheart cherry trees decreased the rain cracking between 31.18% and 44.60%, depending on the level of the surfactant mixture (Tween 80 and Span 80 at a 1:1 ratio) used in the coating [45]. The surfactant mixture was the most critical factor affecting the wettability, hydrophilicity, and elasticity of the coatings [45]. However, in other study an opposite result was found when spraying an anti-transpirant (Vaporgard) on Royal Ann sweet cherry trees 7 d before harvest [46]. They revealed that applying Vaporgard produced more cracking than in the controls by increasing the overall turgor in the trees and causing the cherries to exceed the strength of the cuticle or wall against rupture with minimal water absorption. 

#### 3.3.2. Resistance to Water Vapor Transfer (RWVT)

**Figure 3:**
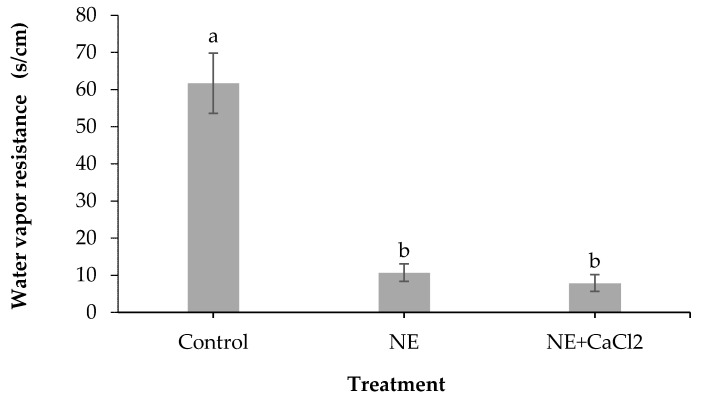
Effect of coating sweet cherries with nanoemulsion (NE) and nanoemulsion plus CaCl_2_ (NE + CaCl_2_) on the resistance to water vapor transfer. (a–b) Different letters indicate significant differences between treatments (*p* < 0.05).

Figure 3 shows the RWVT of coated and uncoated sweet cherries. All the coated fruits were less resistant to water loss than the control (Figure 3). The significant decrease in RWVT in the coated sweet cherries could be due to the emulsifier (Tween 80) that altered some epicuticle sites by changing, partially damaging, or extracting wax from the cuticle. These alterations could cause the dilation of the hydrophilic pores and lead to greater cuticle permeability [39,47]. Crosslinking with CaCl_2_ did not increase the resistance to water loss despite the presence of an additional insoluble cross-linked alginate layer. This behavior could be associated with the swelling of the NE layer produced by water vapor transfer from the inside to the outside of the fruit. This increased volume of the NE layer can cause mechanical damage in the outer layer of cross-linked alginate, thus reducing its water vapor barrier capacity.

### 3.4. Fruit Quality Parameters

#### 3.4.1. Weight Loss

**Figure 4:**
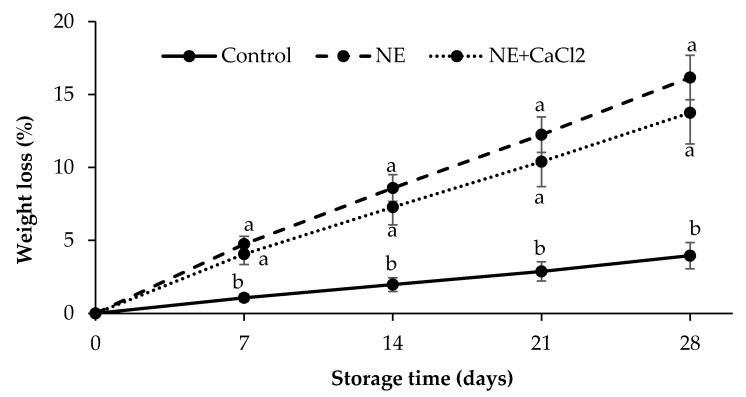
Effect of coting sweet cherries with nanoemulsion (NE) and nanoemulsion plus CaCl_2_ (NE + CaCl_2_) on weight loss during cold storage. (a–b) Different letters indicate significant differences between treatments (*p* < 0.05).

Fruit weight loss during postharvest is due to the gradient of water vapor pressure between the fruit and the surrounding air [48]. Both the layer of epidermal cells and the cuticle are responsible for controlling this weight loss. Sweet cherries are characterized by rapid senescence and a cuticle with low resistance to water vapor diffusion, which promotes rapid water loss from the fruit and stem [49]. In the present study, the weight loss of coated and uncoated sweet cherries progressively increased with storage time (Figure 4). Contrary to the expected effect, coated sweet cherries experienced greater weight loss (16% and 14% after 28 d at 4 °C for cherries coated with NE and NE + CaCl_2_, respectively) than uncoated cherries (4% after 28 d at 4 °C). 

Similar results have been reported by Chiabrando and Giacalone [50] when applying alginate coatings at 1%, 3%, and 5%. These authors found that applying these coatings was not effective to reduce the expected weight loss. When using Big Lory sweet cherries, they obtained 8.15%, 7.40%, and 8.25% weight loss, respectively, after 21 d at 2 °C, while uncoated cherries reached 7.35%. In Grace sweet cherries, the control fruits and those coated with 1% alginate achieved a 10% weight loss, while cherries coated with 3% and 5% alginate reached 12%. However, Díaz-Mula et al. [1] obtained weight losses of 5.93%, 4.88%, and 3.71% in Sweetheart cherries when was applying an alginate coating at 1%, 3%, and 5%, respectively, after 16 d at 2 °C. Uncoated cherries had a weight loss of 6.81%. Some previous studies have indicated that gum arabic, almond gum [51], and chitosan [52] coatings can reduce the weight loss of sweet cherries.

In the present study, the higher weight loss of coated sweet cherries compared with uncoated sweet cherries can be attributed to the low RWVT of coated sweet cherries. This lower barrier capacity was caused by the emulsifier present in the NE coating, which increased the permeability of the sweet cherry cuticle, as described in Section 3.3.2. On the other hand, the crosslinking of the NE coating with CaCl_2_ only slightly reduced the weight loss of the sweet cherries, due to the increase in the water vapor permeability of the alginate layer caused by its swelling. 

#### 3.4.2. Color Attributes

**Figure 5:**
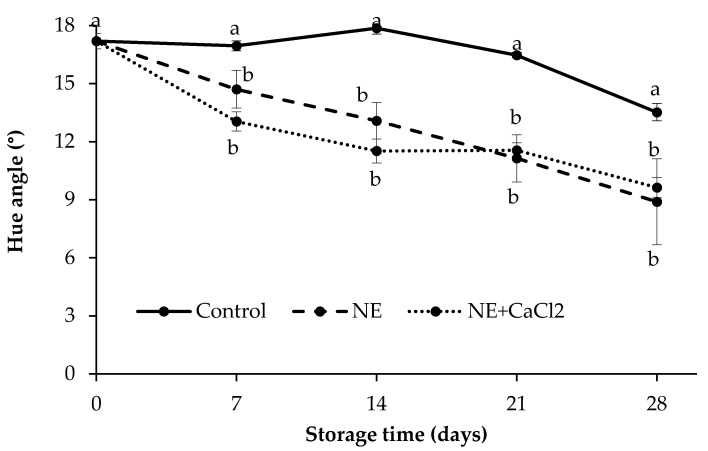
Color evolution (hue angle) of uncoated sweet cherries (control), sweet cherries coated with nanoemulsion (NE) and nanoemulsion plus CaCl_2_ (NE + CaCl_2_) during cold storage. (a–b) Different letters indicate significant differences between treatments (*p* < 0.05).

Changes in the skin color parameters of uncoated and coated sweet cherries during storage are shown in Figure 5. The hue angle was correlated with the anthocyanin content and the lowest hue angle values corresponded to high anthocyanin contents [53]. Hue angle values slightly decreased during storage in all the samples; the reduction was more pronounced from day 21 onward, especially in coated fruits (Figure 5). The coated fruits had lower hue values than uncoated fruits, and there were no significant differences between NE and NE + CaCl_2_. Decreased hue values represent the progress of the fruit ripening process, reaching darker red colors in more advanced stages of maturity. This decrease in hue values during storage has also been described for other sweet cherry cultivars [54] and in sweet cherries coated with alginate [1] and Semperfresh (sucrose esters and mono-diglycerides of fatty acids and sodium carboxymethyl cellulose) [14]. 

The lower hue values of the coated fruits compared with the uncoated fruits can be related to the greater water loss experienced by the coated fruits during storage and promoted by the emulsifier, as discussed in Section 3.4.1. As a consequence, the anthocyanin content in these fruits increased, thus producing a darker red color. In turn, no significant differences were observed in the hue values in the fruits coated with NE and NE + CaCl_2_, mainly due to the fact that the sweet cherries with these coatings presented similar weight losses (Figure 4).

#### 3.4.3. Fruit Firmness

**Figure 6:**
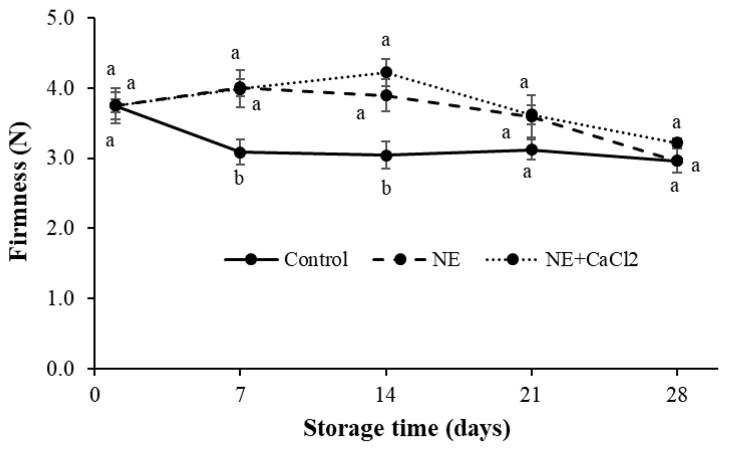
Changes in the firmness of uncoated sweet cherries (control), cherries coated with nanoemulsion (NE), and cherries coated with nanoemulsion and the application of CaCl_2_ (NE + CaCl_2_) during cold storage. (a–b) Different letters indicate significant differences between treatments (*p* < 0.05).

Changes in postharvest firmness can be produced by moisture loss and enzymatic changes [55]. All the coated and uncoated fruits showed decreased firmness during storage (Figure 6). The coated sweet cherries exhibited higher firmness values than the uncoated fruits; however, no significant differences were observed between them as of day 21 of storage. 

Similar results have been described in several studies applying different edible coatings on sweet cherries such as Semperfresh [14], alginate [1,50], almond gum, gum arabic [51], and guar gum with ginseng extract [56]. In these studies, the greater firmness retention of coated sweet cherries has been explained by the delayed enzymatic degradation of the components responsible for fruit structural rigidity caused by a decreased respiratory rate and cold temperature; it is also associated with reduced fruit moisture loss or maintained cellular turgor pressure.

No significant differences in firmness were observed between the fruits coated with NE and NE + CaCl_2_. Even when a crosslinked alginate layer was formed on the surface of sweet cherries, it was not able to improve its water barrier capacity, obtaining a similar weight loss levels as those coated with NE and producing the same mechanical behavior. 

#### 3.4.4. Determination of Titratable Acidity (TA) and Total Soluble Solids (TSS)

**Figure 7:**
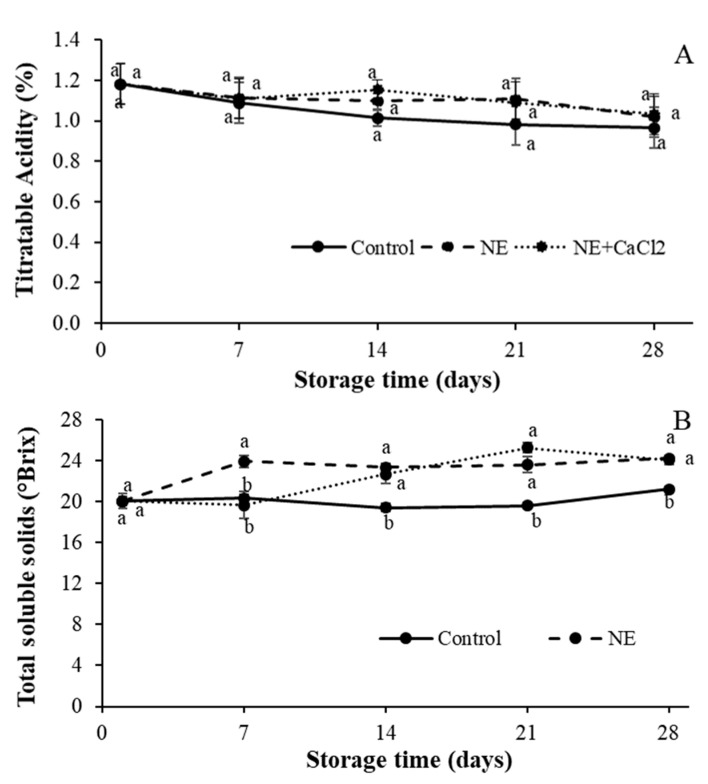
Effects of coating sweet cherries with nanoemulsion (NE) and nanoemulsion plus CaCl_2_ (NE + CaCl_2_) on (**A**) the titratable acidity and (**B**) total soluble solids during cold storage. (a–b) Different letters indicate significant differences between treatments (*p* < 0.05).

The TA and TSS of coated and uncoated sweet cherries are shown in Figure 7. The TA value at harvest was 1.18 ± 0.1% malic acid equivalent, which decreased during storage for all cherries reaching at the end of the storage period values of 0.97 ± 0.02%, 1.02 ± 0.03%, and 1.03 ± 0.04% for control, NE, and NE + CaCl_2_-coated sweet cherries, respectively (Figure 7A). The TA value decreased over time (Figure 7A) because organic acids are substrates for the enzymatic reactions of respiration [57]. From day 7 onwards, the uncoated sweet cherries showed a greater reduction in TA than the coated sweet cherries; however, these differences were not significant. These results indicate that the coatings used were not able to significantly reduce the respiratory rate of fruits because they did not delay the use of organic acids, which are used in the enzymatic reactions of respiration [14]. Similar results have also been described when using 1% alginate coatings [50] and coatings of different types of 1% chitosan [58] in sweet cherries. 

Additionally, no significant differences were observed between the NE and NE + CaCl_2_ coatings. These results would also indicate that the formation of a cross-linked layer of alginate fails to improve the gas barrier capacity of the coating and thus reduce the respiratory rate of the coated fruits. Possibly, the nanoemulsified coating and its cross-linked layer undergo a plasticization process as water vapor is lost to the environment, reducing the gas barrier capacity of these coatings.

The initial TSS value was 20.0° Brix, but it increased during the storage period (Figure 7B). The coated sweet cherries exhibited a greater increase in TSS than the uncoated fruits, reaching values of 24.1 and 21.20° Brix, respectively, at the end of storage. The application of edible coatings on sweet cherries usually produces a delayed ripening process and a low increase in TSS compared with uncoated sweet cherries [54,55]. However, the opposite result was obtained in the present work. The higher TSS values for coated sweet cherries can be largely explained by the higher water loss in these fruits. Several authors point out that the loss of water during storage produces the concentration of sugars in coated fruits [55,59]. In the present study, the greater weight loss shown by the coated fruits compared to the uncoated fruits (Figure 4), due to the presence of emulsifier in the NE coatings as described in Section 3.3.2 and Section 3.4.1, would be the main cause of these higher TSS values. This is also the reason why no significant differences were detected between the fruits coated with NE and NE + CaCl_2_, since the fruits with both coatings showed similar levels of weight loss. As previously mentioned, the formation of an additional layer of cross-linked alginate on the sweet cherries was not able to reduce their water loss, because the nanoemulsified coating undergoes a swelling process during storage, reducing its water vapor barrier capacity.

The sourness and sweetness of sweet cherries are important for consumer acceptance. The TSS can be used to measure sweetness and TA to measure sourness [60]. Crisosto et al. [61] indicated that consumer acceptance and the level of satisfaction with sweet cherries increased with higher acidity (TA > 0.80%) and sweetness (TSS > 20.0%). According to our results, coated sweet cherries could have better consumer acceptance and level of satisfaction than the uncoated sweet cherries after 14 d because they had higher TA and TSS values.

### 3.5. Total Soluble Phenolics (TSP), Anthocyanin Content, and Antioxidant Capacity (AC) 

The consumption of fruit and vegetables with high phytochemical contents such as anthocyanins and other polyphenolics, carotenoids, and vitamins C and D have been associated with the prevention of different chronic diseases [62]. Phenolic compounds also contribute to the sensory and organoleptic quality of sweet cherries, such as flavor and astringency [2] and their antioxidant potential [54].

**Figure 8:**
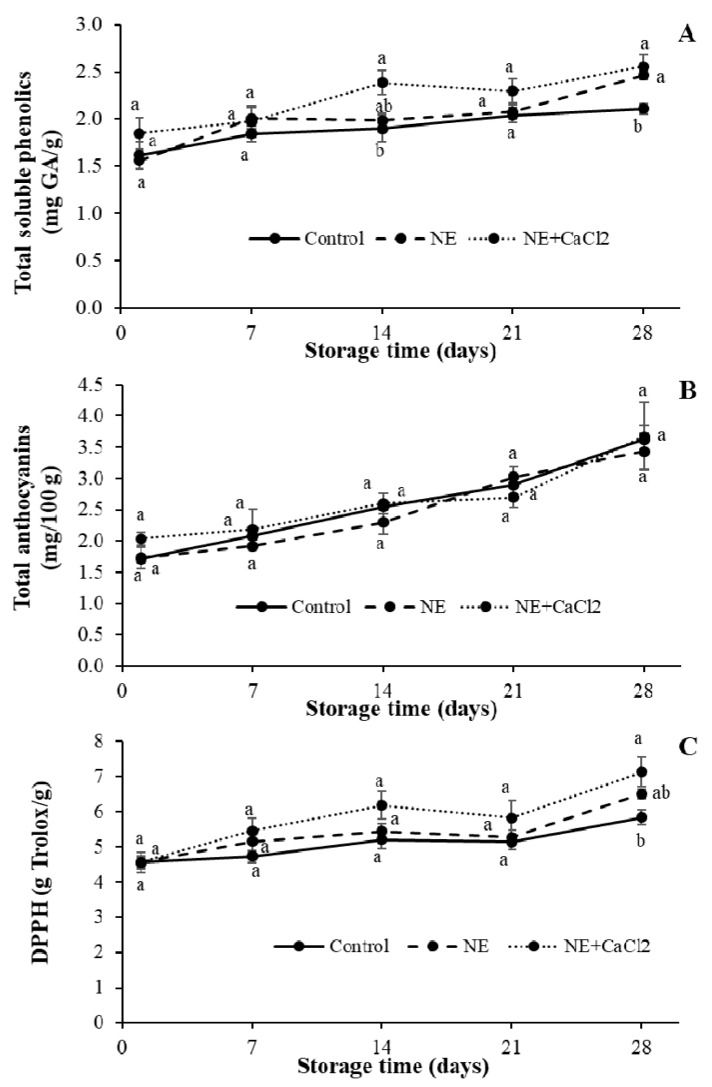
Effects of coating sweet cherries with nanoemulsion (NE) and nanoemulsion plus CaCl_2_ (NE + CaCl_2_) on (**A**) total soluble phenolics, (**B**) total anthocyanins, and (**C**) antioxidant capacity (DDPH) during cold storage. (a–b) Different letters indicate significant differences between treatments (*p* < 0.05).

Figure 8 shows that coatings affected the TSP content and AC of sweet cherries but not their anthocyanin content. The initial TSP content in uncoated sweet cherries was 1.62 ± 0.25 mg GAE/g (Figure 8A). The TSP values of uncoated and coated sweet cherries progressively increased over time. Coated sweet cherries had higher TSP values than uncoated sweet cherries. The TSP value after 28 d for uncoated sweet cherries was 2.11 ± 0.10 mg GAE/g, whereas the TSP values for sweet cherries coated with NE and NE + CaCl_2_ were 2.47 ± 0.08 and 2.56 ± 0.22 mg GAE/g, respectively. 

The increase in the total polyphenol content in sweet cherries in the present study was contrary to the expected results. Several studies have reported a decrease in the total polyphenol content in sweet cherries during storage as a result of peroxidase and polyphenol oxidase enzyme activity during the ripening process. They have also mentioned that applying edible coatings on sweet cherries has achieved a higher total polyphenol retention compared with uncoated fruits due to the formation of a protective barrier to gases on their surface [49,50,63]. 

In our study, the increased total polyphenol content can be explained by the development of two phenomena. First, the formation of a high gas barrier coating might have reduced phenol enzymatic oxidation. Second, the significant water loss in the coated fruits during storage due to the emulsifier in the coatings might have produced a concentration of the soluble polyphenolic compounds.

Anthocyanins are responsible for the red color in sweet cherries; their content increases during postharvest storage because the ripening process progresses and they are used as a quality indicator of cherries [64]. The anthocyanin content for all sweet cherries progressively increased during the storage period, and there were no significant differences between the coated and uncoated fruits (Figure 8B). These results were consistent with the decrease in hue angles observed in sweet cherries during the same period (Figure 5), which indicates a color change in sweet cherries from reddish red to more violet red [53,54]. However, even when the coated fruits had lower hue values than the uncoated fruits, it was not possible to detect these differences in anthocyanin content, which likely occurred because of the increased anthocyanin concentration in these coated fruits caused by significant water loss during storage.

The AC values of stored uncoated and coated sweet cherries progressively increased over time (Figure 8C). In general, sweet cherries coated with NE + CaCl_2_ exhibited a higher antioxidant activity than uncoated sweet cherries and those coated with NE. At the end of storage, the inhibition of DPPH radicals was 7.13 ± 0.74, 6.50 ± 0.24, and 5.83 ± 0.37 g Trolox/g for NE + CaCl_2_, NE, and uncoated sweet cherries, respectively. The higher CA of the coated fruits compared with the uncoated fruits could be explained by the increase in the TSP due to the modified internal atmosphere caused by the coatings [63] and the water loss indicated in Section 3.4.1.

## 4. Conclusions

The results presented in this study showed that the nanoemulsified coatings based on alginate and soybean oil presented different effects on the barrier properties and quality parameters of sweet cherries. The NE + CaCl_2_ coatings were able to significantly reduce the cracking of sweet cherries, achieving a 53.3% reduction compared to the control fruits, due to the formation of a cross-linked layer on the surface of the coatings caused by the addition of CaCl_2_ as a cross-linking agent.

The Ne coatings had a limited effect on the delay of the ripening process and the quality parameters of the cherries. The presence of an emulsifier in these coating could have altered the cuticle waxes and caused increased weight loss in sweet cherries coated, reducing their potential effect on the quality parameters of the cherries. This behavior was not improved either with the formation of the cross-linked alginate layer (NE + CaCl_2_). However, a higher retention of total polyphenols and antioxidant capacity of the sweet cherries coated with NE + CaCl_2_ was verified. Future studies should focus on optimizing the amount of emulsifier in nanoemulsified coatings to improve the barrier properties and make them more effective in delaying the ripening of sweet cherries.

## Data Availability

Not applicable.
